# Inhibition Ability of Natural Compounds on Receptor-Binding Domain of SARS-CoV2: An In Silico Approach

**DOI:** 10.3390/ph14121328

**Published:** 2021-12-18

**Authors:** Miroslava Nedyalkova, Mahdi Vasighi, Subrahmanyam Sappati, Anmol Kumar, Sergio Madurga, Vasil Simeonov

**Affiliations:** 1 Inorganic Chemistry Department, Faculty of Chemistry and Pharmacy “St Kliment Ohridski”, University of Sofia, 1164 Sofia, Bulgaria; 2 Department of Chemistry, University of Fribourg, 1700 Fribourg, Switzerland; 3 Department of Computer Science and Information Technology, Institute for Advanced Studies in Basic Sciences (IASBS), Zanjan 45137-66731, Iran; vasighi@iasbs.ac.ir; 4 Raman Research Institute, C. V. Raman Avenue, Bengaluru 560012, India; ssappati@iisertvm.ac.in; 5 Department of Pharmaceutical Sciences, School of Pharmacy, University of Maryland, Baltimore, MD 21201, USA; anmol@outerbanks.umaryland.edu; 6 Department of Material Science and Physical Chemistry & Research Institute of Theoretical and Computational Chemistry (IQTCUB), University of Barcelona, 08007 Barcelona, Spain; s.madurga@ub.edu; 7 Analytical Chemistry Department, Faculty of Chemistry and Pharmacy “St Kliment Ohridski”, University of Sofia, 1164 Sofia, Bulgaria; vsimeonov@chem.uni-sofia.bg

**Keywords:** SARS-CoV-2: RBD, natural compounds, docking, machine learning, computer-aided drug design, molecular dynamics (MD) simulations

## Abstract

The lack of medication to treat COVID-19 is still an obstacle that needs to be addressed by all possible scientific approaches. It is essential to design newer drugs with varied approaches. A receptor-binding domain (RBD) is a key part of SARS-CoV-2 virus, located on its surface, that allows it to dock to ACE2 receptors present on human cells, which is followed by admission of virus into cells, and thus infection is triggered. Specific receptor-binding domains on the spike protein play a pivotal role in binding to the receptor. In this regard, the in silico method plays an important role, as it is more rapid and cost effective than the trial and error methods using experimental studies. A combination of virtual screening, molecular docking, molecular simulations and machine learning techniques are applied on a library of natural compounds to identify ligands that show significant binding affinity at the hydrophobic pocket of the RBD. A list of ligands with high binding affinity was obtained using molecular docking and molecular dynamics (MD) simulations for protein–ligand complexes. Machine learning (ML) classification schemes have been applied to obtain features of ligands and important descriptors, which help in identification of better binding ligands. A plethora of descriptors were used for training the self-organizing map algorithm. The model brings out descriptors important for protein–ligand interactions.

## 1. Introduction

The present worldwide pandemic caused by the SARS-CoV-2 strain of SARS coronavirus requires conventional and non-conventional methods of curing it. The COVID-19 virus is largely intractable to currently available antivirals. Nonetheless, the effective life span of most antivirals is limited. The application of alternative drugs, e.g., phytocompounds, can provide an auxiliary treatment plan, owing to their abundance, ease of accessibility, and low toxicity. Phytochemicals are secondary metabolites produced by plants for their survival and propagation. Various phytocompounds have been validated for their antiviral activity. Usually, the information about the healing ability of ethnomedicines is largely observational rather than strictly scientific. Phyto-therapeutic agents have gathered increased attention from the scientific community to deal with the current pandemic. In a recent article by Swain et al. [1], a long list of potential anti-Covid phytochemicals was published along with the plant source and the inhibition effects.

The process of developing new drugs is a long-lasting and costly effort. The chemical diversity, unique properties, and wide structural variety make the natural compounds an excellent initial point serving as a good template for new drug discovery. Natural products with antiviral activity may provide an auxiliary way for tackling SARS-CoV-2 infections. In this study, we investigated the antiviral activity of flavonoids against SARS-CoV-2 [2].

The aid of computational drug design methods opens the possibilities of transforming natural products for many health issues. The development and comprehension of antiviral drugs have led to a wide range of natural compound studies as an efficient and effective strategy, even with the aid of nanotechnology approaches [3,4], for identifying effective COVID-19 medication. To explore novel and potent therapies, a fundamental understanding of the interplay of molecular forces involving the spike protein and potential inhibitors in an aqueous medium is critical. The class of flavonoids, terpenes, terpenoids, alkaloids, quinone derivatives, and esters was less studied for drug interactions, and this class consists of hydrophobic and hydrophilic constituents. Among natural compounds, flavonoids constitute a possible target for antiviral drugs due to their large spectrum of medicinal properties (antioxidant, anti-inflammatory, and antiviral). Some flavonoids with substantial antiviral activity against SARS-CoV-2 are kaempferol, quercetin, myricetin, fisetin, and derivatives. The efficacy of many dietary flavanols as potential antiviral drugs for SARS-CoV-2 enzymes was performed by Patel et al. using in silico assays and machine learning techniques [5]. Several other articles have studied the effectiveness of natural compounds in blocking the binding of the spike protein with the angiotensin-converting enzyme 2 (ACE2) receptor present on human cells [6,7,8,9,10,11]. Recent advances in machine learning (ML) methods have created the ability to identify new natural compounds for a given target [12,13]. A variety of machine learning algorithms was applied recently to resolve these problems to boost and reveal the treatment capabilities of natural compounds [14,15,16,17]. A recent paper by Barazorda-Ccahuana et al. [18] showed significant effort in implementing computational methods to determine the efficacy of molecular inhibitors in different protonation states at different pHs and their applications in therapeutics.

In the recently published paper by Qiang et al. [19], the ML model was developed on a natural product dataset obtained using the ChEMBL dataset. The transfer learning algorithm, data balancing technique, and model diagnostics applied in their work show a promising result in determining the lead compound. Glycyrrhizin and Nafamostat mesilate were selected as candidates for drug delivery. These compounds were transferred into micelle nanoparticles to improve the stability and availability of drugs in the cells to treat COVID-19. Such a target prediction model can be applied in natural product-based drug discovery to find more lead compounds and assist in drug repurposing. The free energy evaluations predict the missing experimental data for those receptor–ligand complexes with the absent experimental study [20]. The data thus obtained can be adapted for building a training algorithm. The computationally obtained data could indeed decrease the reliability level of the training data set. However, the new prediction algorithms have a higher threshold for minimizing errors.

Last year, all available resources and ML were forced to narrow down the drug candidates and minimize clinical trial failure. Kowalewski and Ray developed models to help identify effective drugs against SARS-CoV-2 proteins. They collected 14 million chemicals from ZINC databases. A partitioning algorithm that follows the proper descriptor space was also explored [21]. In silico modeling has been recognized as a path for applying natural products as potential disruptors of the initial infection. Experimental findings also exist in conjunction with in silico studies, which show the potential of plant-originated therapeutics for the treatment of COVID-19 [22].

The literature still lacks binding abilities and the dynamic behavior of many natural compounds. This study presented an in silico drug design strategy based on docking, MD simulations and combined with ML methods with a broad descriptors database generated by [23] to examine readily available natural-based combinations. The spike protein is responsible for invading the virion into the host cell by binding to the cell surface via the ACE2 receptor. Salt bridges and hydrogen bonding stabilize this interaction between the receptor–binding domain in the S1 subunit of the spike protein (S1-RBD) of SARS-CoV-2 and the ACE2. In this paper, 40 prevalent phytochemicals were studied for their binding affinity. The database of natural ligands was targeted to the available X-ray crystal structure of the SARS-CoV-2 S1-RBD bound to ACE2 (PDB ID: 6M0J) for docking. Their derivatives are polyphenols, flavonoids, alkaloids, terpenes, diarylheptanoids, and lectins. These compounds are polyphenols and flavonoids, alkaloids, anthraquinones, saponins, terpenes, coumarins, diarylheptanoids, and lectins. Some of the selected candidates from natural products targeted to the viral RBD of the SARS-CoV-2 spike protein have previously been studied using isothermal titration calorimetry ITC binding assay [24], viz. celastrol, saikosaponin C, and amentoflavone. The dual computational approach and classification (supervised and unsupervised) methods applied in this work indicate that amentoflavone and glyrimizine may disrupt the interaction between ACE2 receptors and the spike protein of SARS-CoV-2. These natural products may bind to the viral spike protein, preventing SARS-CoV-2 from entering cells.

A sequence of methodologies was employed to establish the nature of the spike protein, stability of ligand-binding to spike protein, and the dominant characteristics required for antagonistic behavior of ligands. The best candidates’ ranking is based on a binding score and is proven by molecular dynamics simulations, followed by a classification algorithm and dimensionality reduction approach as principal component analysis (PCA) and a self-organizing map (SOM). The docking energies and geometrical properties between a ligand–receptor complex of a 40-natural-compound library are shown in Supporting Information (Supporting Information Table S1 Dataset of 40 natural compounds with docking scores).

## 2. Results and Discussion

### 2.1. Molecular Docking Results

**Figure 1:**
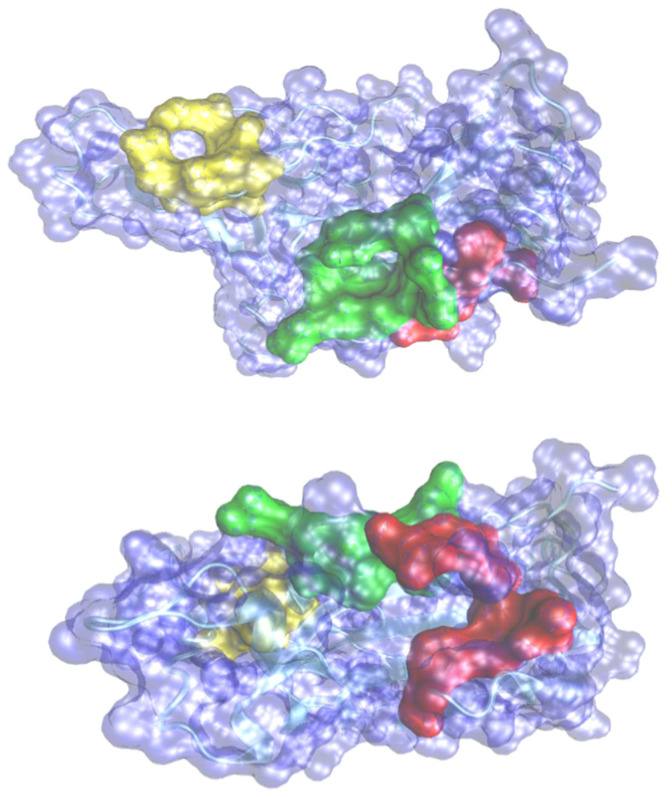
Predicted pockets in two different perspectives of views of the representation of the surface of spike fragment with the three pockets as indicated in Table 1. Pocket 1 in red, Pocket 2 in yellow and pocket 3 in green.

The primary approach in structure-based drug design is to use in silico virtual screening methods to screen databases of small molecule compounds against targets of interest. In the current article, molecular docking software, Auto Dock Vina was employed to identify potential phytotherapeutic binders toward the receptor-binding domain (RBD) (PBD: 6M0J) of the SARS-CoV-2 spike protein. AutoDockVina software is now integrated into the SAMSON molecular design platform (https://www.samson-connect.net, accessed on 21 November 2021) as a SAMSON extension. The extension provides additional functionality to easily prepare receptors and ligands, dock ligand libraries, analyses and export docking results. We used SAMSON and the Vina extension to configure calculations, export input files, run docking calculations in the cloud, and import results for visualization and analysis. The search space was defined by a docking box wrapper, the space around the receptor that will be searched according to the selected pocket for the RBD. The size of the grid box was set to 44.4, 21.3, 55.3Å for each axis. The number of modes was set to 200 and the exhaustiveness was set to 10. The active pocket amino acid residues were predicted by the webserver CASTp [23] and summarized in Table 1 and depicted in Figure 1. A total of 40 molecules with 25 flexible chains for docking calculations was performed. Binding energy and the constant inhibition were obtained for 200 poses for each molecule. A novel protein–ligand interaction analyzer tool available with SAMSON software was used to understand the stability of the protein–ligand complexes interaction based on hydrogen bond analyses, amino acids environment, histogram distribution of the amino acids, SASA (solvent accessible surface area), radius of gyration, and contact area receptor–ligand.

### 2.2. Molecular Dynamics Simulation Protocol

The additive forcefield parameters for the ligands were obtained using CHARMM General Force Field (CGenFF) online web server (https://cgenff.umaryland.edu, accessed on 21 November 2021) and were later converted to GROMACS force field format. The structure of the receptor-binding domain (6M0J) of the SARS-CoV-2 spike protein was obtained from the Protein Data Bank (PDB), and topology was prepared using CHARMM36 force field. We used the docked conformation to generate the coordinates of protein-drug complex. The combined protein and drug (system obtained from the autodocking studies as an initial guess) was kept in a box of dimension 90 × 90 × 90 Å^3^ and solvated with the SPC/E water molecule. Physiological ion concentrations of Na^+^ and Cl^−^ were used to neutralize the system.

Initially, each system was energy minimized using the steepest descent method for 10,000 steps, followed by heating it to 300 K in 1 ns using a Berendsen thermostat with a coupling constant of 0.6 ps. We applied restraints of 25 kcal/mol/Å^2^ on heavy atoms of proteins during the heating process. Thereafter, equilibration was carried out for 10 ns at constant temperature (300 K) and pressure (1 bar) without any restraints using the same Berenson thermostat and barostat with coupling constants of 0.6 ps each. Finally, we performed 300 ns unrestrained NPT equilibration using the Nosé-Hoover thermostat with a coupling constant of 0.6 ps. During the simulation, all atoms were constrained using Linear Constraint Solver (LINCS), and Particle Mesh Ewald (PME) method was used for electrostatics with long-range cut-off of 10 Å. The distance cut-offs for the van der Waals (vdW) were kept at 10 Å. The time step for each simulation was taken to be 2 fs.

### 2.3. Docking Results

**Figure 2:**
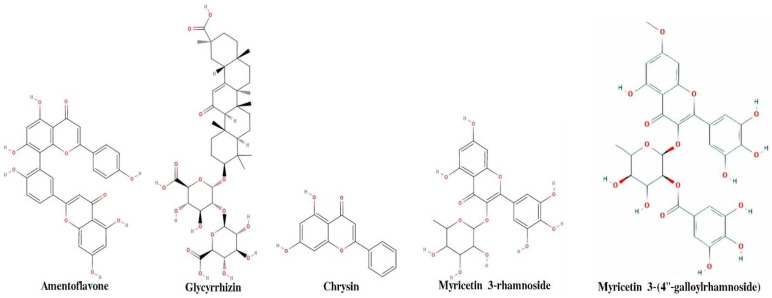
2-D representation of five ligands that show maximum binding affinity with 6M0J.

Structure-based drug discovery efforts require knowledge of drug-binding sites on target proteins. The above procedure generates a set of residues that are part of the three identified pockets. To investigate which predicted sites are likely to be druggable, molecular docking is performed on each site using 40 molecules. The dock score (presented in the tables in Supporting Information) is used here to verify and expand upon the ML predictions based on a combination of a descriptors obtained using AlvaDesc v.2 software (Milano, Italy https://www.alvascience.com/alvadesc/, accessed on 15 October 2020). It is expected that predictions are more likely to be accurate when the residues with high binding scores are in the same region as residues with high dock scores. The residues were further classified using ML methods to bring out broad set of features that are helpful to identify druggable quality of natural compounds (complete dataset with a full list with descriptors is mentioned in the SI). Figure 2 shows two-dimensional structure of five compounds, which shows maximum binding affinity in molecular docking. Drug-like properties of top five compounds are shown in Table 2. According to Lipinski’s rule, a compound is labeled as drug-like when it meets the following criteria: molecular weight < 500 Da, H bond donor ≤ 5, H bond acceptor ≤ 10, Log *p* ≤ 5, and TPSA > 140 Å^2^. Table 3 shows analysis of molecular docking results of these top five compounds.

**Figure 3:**
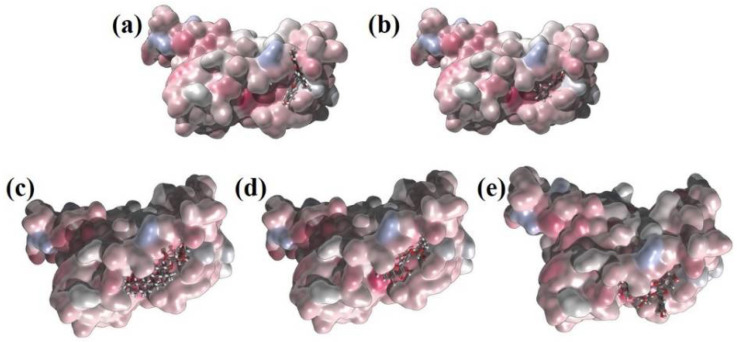
Representation of the surface charge distribution localization for: (**a**) Amentoflavone; (**b**) Chrysin; (**c**) Glycyrrhizin; (**d**) Myricetin_3′-Rhamnoside; (**e**) Myricetin_3-(4″-Galloylrhamnoside) with line representation and RBD with surface representation using atom charge scale (red-blue color palette changes from negative (blue) through neutral (white) to positive (red).

**Figure 4:**
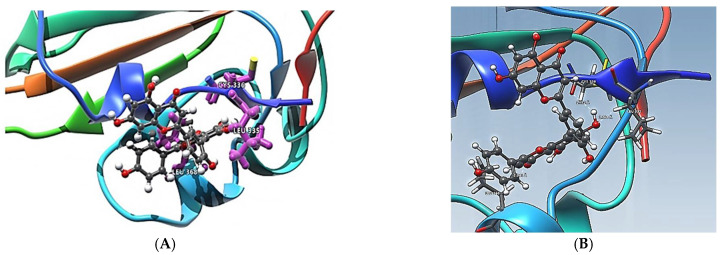
(**A**) Amentoflavone complexes with RDB. Docking pose for Amentoflavone showing some closed localized amino acids (LEU 335, 368 and CYS 336) of its environment; (**B**) Hydrogen-acceptor and hydrogen-donor distances for Amentoflavone with LEU 335, 368 and CYS 336.

The first five candidate molecules from the docking study are depicted in Table 3 for inhibition candidates of the spike RBD protein with binding energies ranging from −9 to −8.3 kcal/mol. In Figure 3, each of the five residues bound to the protein are shown. Hydrophobic Gaussian surface was used for graphical representation of the protein. The 40 phytocompounds were analyzed for the binding affinity. Figure 4 shows interactions of Amentoflavone and S-protein as obtained using molecular docking. The amino acid residues environment for the Amentoflavone shows effective hydrogen bond formation with LEU 335, CYS 336, and LEU 368. The obtained data for the hydrogen bonds based on the docking suggested that the interactions of the specific residues (amino acids residues) of RBD of SARS-CoV-2 S-protein and the corresponding ligand are capable of forming a stabilized hydrogen bond. LEU 335 is a part of a coil structure of the RDB, and LEU 368 is a part of a helix structure. Out of the 13 ligand-interacting residues, six are strongly hydrophilic, and the remaining seven are hydrophobic. These amino acids formed H bond with the ligand. The hydrophilic residues around the ligand create a hydrophobic environment and facilitate the binding to the hydrophobic pocket.

The radius of gyration is used to analyze the effect ligand on the distortion of the conformation of RBD upon binding. It could be seen that the RBD does not change significantly in terms of Rg for any of the shown ligands. This depicts no significant change in the protein conformation before and after ligand docking. SASA is another metric that allows us to determine the protein’s flexibility, stability, and folding in the presence and absence of ligands. With this property, we have a clearer picture of the immediate changes in the protein conformation. Since the values obtained for SASA between the complex RBD and the inhibitor candidates were without a significant deviation, we can conclude that the globally available surface area is preserved before and after the docking.

### 2.4. Molecular Dynamics Simulations

In order to prove the obtained docking scores and bring out the interaction between the class of flavonoids, terpenes and terpenoids with the spike protein, we performed all atom MD simulations on protein–ligand elected conformations. Four of the complexes exhibiting best docking energies and three of low binding energies complexes were chosen to figure out the key to successful binding. Preliminary simulations over 300 ns time were performed on the apo protein system and then compared our results with the seven selected protein–ligand complexes. Last 250 ns are considered for all the analysis. During the protein–ligand simulations, a new pocket was identified adjacent to pocket 1 (pocket 1A), showing greater affinity toward some of the ligands (see Table 1).

**Figure 5:**
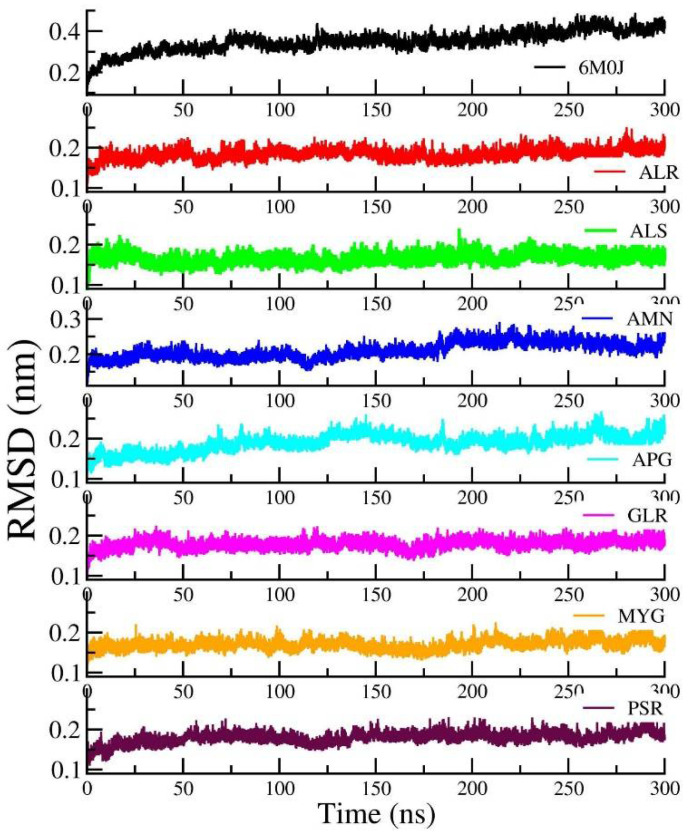
Time dependence of the root mean square deviation (RMSD) of the protein from the production run of all seven protein–ligand complexes and compared with protein in water simulation.

**Figure 6:**
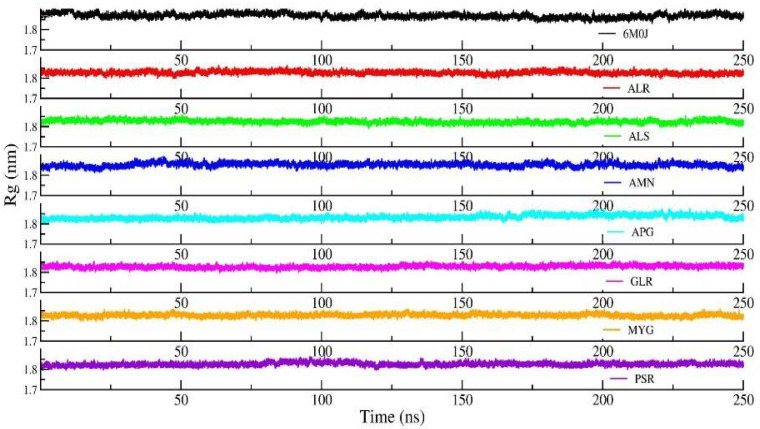
Radius of gyration (Rg) of backbone of the protein from the production run of all seven protein–ligand complexes and compared with protein in water simulation.

**Figure 7:**
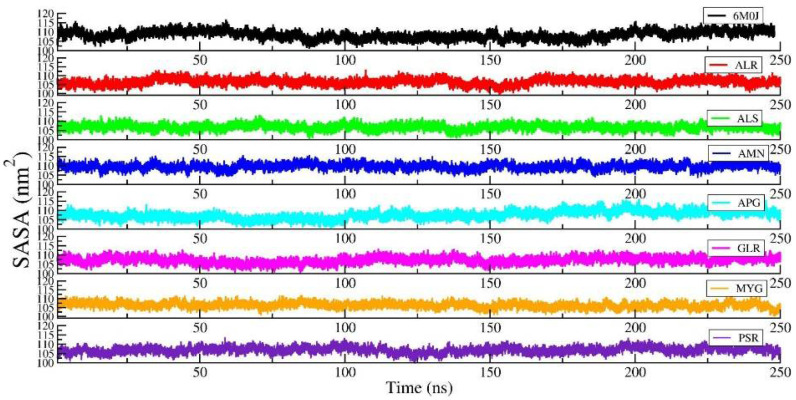
The SASA of the protein in the protein–ligand complex and compared with the protein–water system.

Figure 5 shows the RMSD of the RBD protein in water and compared with protein in all other seven protein–drug complexes. We observed that the protein–water and protein–ligand systems are stable throughout the production run. The RMSD of the protein in the protein–water complex is 0.377 nm. The RMSD of the protein in the protein–Amentoflavone is closer to the protein–water complex. To monitor the change in the structure of SARS-CoV-2 RBD resulting from its interaction with the seven drug molecules, we measured the radius of gyration of the backbone of the RBD as a function of time. Figure 6 shows the Rg of the backbone (BB) of the RBD protein. Furthermore, the average value of Rg of the BB-RBD shows 1.869 nm. It shows that the Rg of the BB-RDB in the protein-Amentoflavone complex is perturbed from the other average Rg values of the protein–ligand complexes. The average SASA of the protein in the protein–ligand complex is 108 nm^2^. However, in case of protein–Amentoflavone complex, average SASA is higher by 2 nm^2^ (see Figure 7).

### 2.5. Hydrogen Bond Interactions between Ligands and SARS-CoV-2

**Figure 8:**
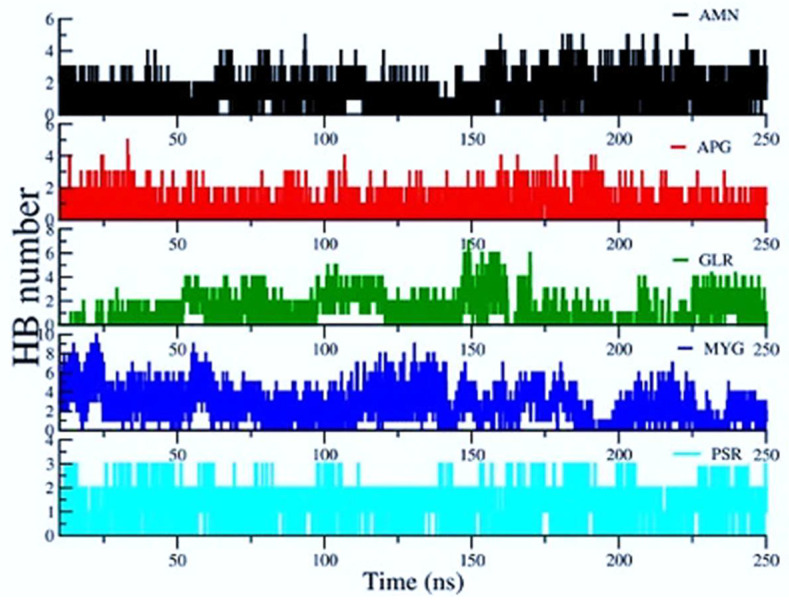
Hydrogen bonds (HBs) between ligands and protein residues.

The H bonded interactions were assessed between polar groups of the drugs and amino acids of the protein. Ligand ALS and ALR did not show any significant H bond contacts. Figure 8 shows the average number of H Bonds between the protein and ligands. We observed that Myricetin-3-(4″-Galloylrhamnoside) creates the highest average number of H Bonds (2.473) with the RBD protein over the entire simulation followed by Amentoflavone and Glycyrrhizin.

**Figure 9:**
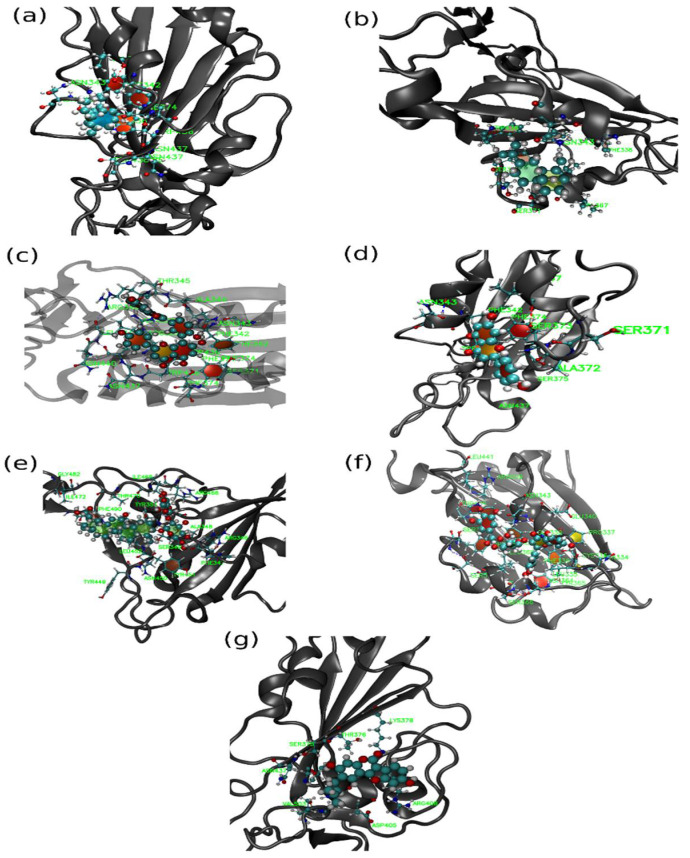
Representative snapshot of protein with (**a**) ALR, (**b**) ALS, (**c**) AMN, (**d**) APG, (**e**) GLR, (**f**) MYG and (**g**) PSR ligands. Here, solid spheres represent drug molecules, CPK model represents residues of protein, which are within 5 Å of the ligand and cartoon (grey) representation for protein.

**Figure 10:**
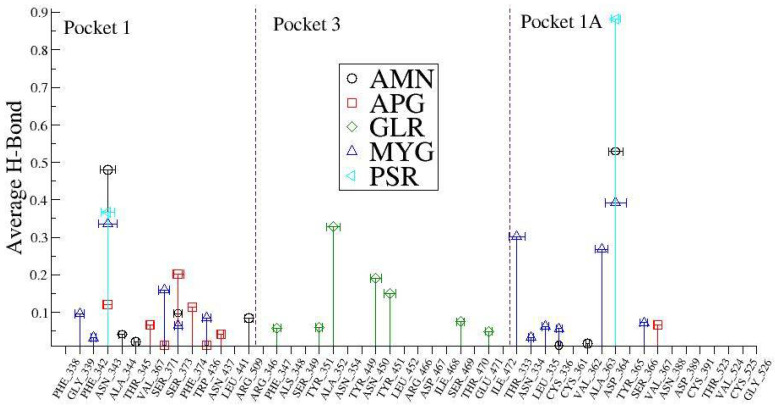
The average number of hydrogen bonds (standard deviation) between the drugs and SARS-CoV-2 spike protein receptor binding domain RBD.

In order to understand these interactions with the natural compounds (Figure 9), we plotted the average hydrogen bonding interactions in pocket 1, pocket 3, and pocket 1A (see Figure 10). We found that asparagine (ASN_343) amino acid makes a strong H bond with the ligand molecules like AMN, PSR, MYG, and APG in the pocket 1. Similarly, other amino acid residues that have hydrogen bonding with the ligands were found to be SER_373, PHE_374, SER_371, VAL_367, GLY_339, ASN_437, ARG_509, ALA_344, THR_345 and PHE_342. Further, in pocket 3, the ligand molecule GLR showed hydrogen bonding with the amino acids at TYR_353, TYR_449, ASN_459, ILE_468, THR_470, SER_349 and ARG_346.

### 2.6. Retention Time of Ligands in Different Binding Pockets of SARS-CoV-2

We investigated the interatomic contacts between ligands and the closest amino acids in the protein across the MD trajectories. We found three important pockets based on the closest distance criteria of the dynamic motion of the ligand. These pockets are similar to the results obtained from molecular docking. We found that pocket 1 and pocket 3 play a crucial role for the selected ligands. Furthermore, we investigated pocket 1A, which is adjacent (and within 1–2 nm) to pocket 1, also playing an important role in our MD simulations.

**Figure 11:**
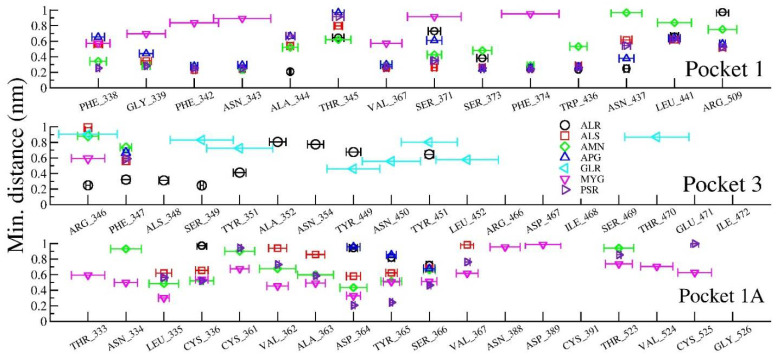
Minimum distance between amino acids of the protein and selected ligands.

To bring out the retention time of ligand in the pockets, the distance between ligands and closest amino acids of the three pockets (viz, in between spiral α-helices, loop dominant region, and at N- or C- terminal of the protein) is plotted in Figure 11. Figure 11 shows the interaction distances and retention time of seven drugs in pocket 1, pocket 1-A and pocket 3. Consistent with our docking results, we found that ALS (Allo-Aromadendrene), AMN (Amentophlavone), APG (Apigenin) and PSR (Psoralidin) majorly stay in pocket 1, while GLR majorly occupies pocket 3 and MYG stays in pocket 1A. Ligand ALR binds in both pocket 1 and pocket 3. However, we noticed that none of the seven ligands are close to the pocket 2, consistent with molecular docking results.

The ligand molecules such as ALR, PSR, ALS, AMN lie within 5Å from the residue in pocket 1, and the ligand molecules such as ALR and GLR bind to the residue within 5Å from pocket 3. Here, ligand molecule PSR is found within a distance of 5Å, indicating the strength of binding of those with the protein. The ligand GLR is found to be away from both pocket 1 and pocket 1A, resulting in its unbound character with the protein active sites.

**Figure 12:**
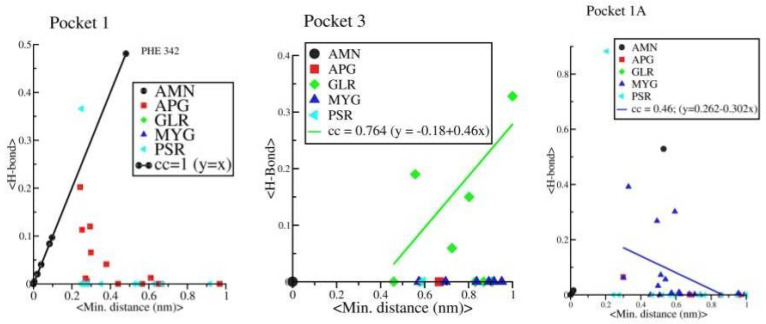
Correlation between average minimum distance between amino acid residues of the protein and ligand vs. the average HB distance between amino acid residues of the protein and ligand.

Further, we observed a linear correlation between minimum distance and average H bonds in protein-AMN complex which indicates that complexation is mainly driven by H bond formations. However, protein-GLR and protein-MYG complexes show poor correlation (see Figure 12). Other four complexes are not showing any such linear correlations. The strength of H bond, strong correlation with minimum distance and long retention time in pocket 1 shows that AMN is the best ligand among all other seven selected ligands that can show antagonistic activity against spike protein, followed by GLR and MYG. The major binding site of AMN, GLR, and MYG are pocket 1, pocket 3, and pocket 1A, respectively.

### 2.7. Hierarchical Clustering

After identification of best ligands that could potentially block the binding of spike protein, we applied ML techniques to identify the features responsible for better binding of these ligands. This can be helpful to identify new molecules with similar features which can show binding with spike protein. All 40 ligands were used for this study. It was of substantial interest to check if the previous partitioning results (partitioning by the use of three different groups of descriptors, namely drug-likeness descriptors, topological and geometric descriptors, and WHIM descriptors) could be confirmed by a coarser scheme of separation (partitioning) offered by hierarchical or non-hierarchical (K-means) cluster analysis. This study has shown that they could be reliably used for this goal. The scheme using PCA and K-means partitioning has led to the following general outputs:

- Drug-likeness descriptors: 10 clusters were identified based on drug-likeness descriptors, wherein GLR and one of the myrecitin ligand fall in one cluster; AMN falls in cluster 8, and the other three myrecitin ligands are distributed in cluster 6 (one ligand) and cluster 9 (2 ligands).
- WHIM descriptors: seven clusters were identified where GLR is in cluster 2; AMN and all myrecitin ligands are in cluster 5;
- Topological and geometrical descriptors: three clusters were identified where GLR and all myrecitin ligands are in cluster 1; AMN is in cluster 2.

**Figure 13:**
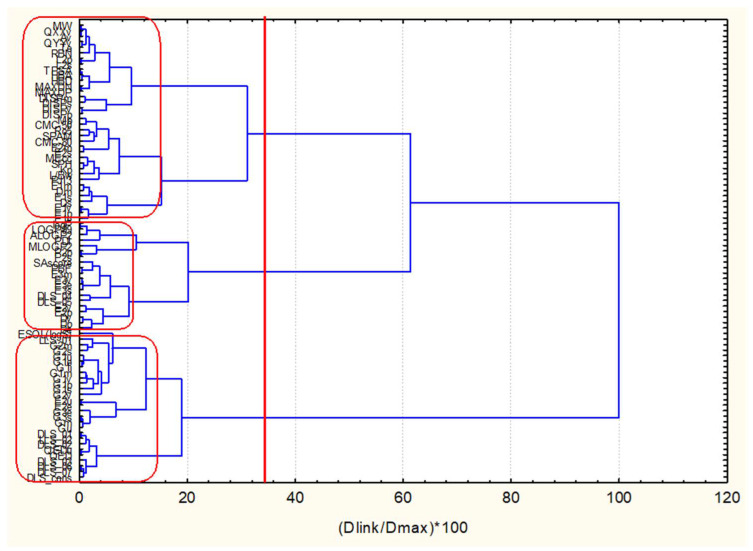
Hierarchical dendrogram for clustering of 83 descriptors.

In Figure 13, the hierarchical dendrogram for clustering of 83 descriptors is shown. Three clusters are identified at significance level 1/3Dmax, i.e., cluster with GLR, cluster with all four myricetins, cluster with AMN. Thus, complete separation is achieved. Considering features of five ligands identified above (see Table 2) to be important for binding activity, the most important descriptors are: Mp, logP and SPAM descriptors (two drug-likeness and one topological descriptor). The results of the graphical representation of the clustering are shown in SI—Hierarchical clustering of 40 ligands.

### 2.8. Hierarchical Clustering of Drug Candidates

**Figure 14:**
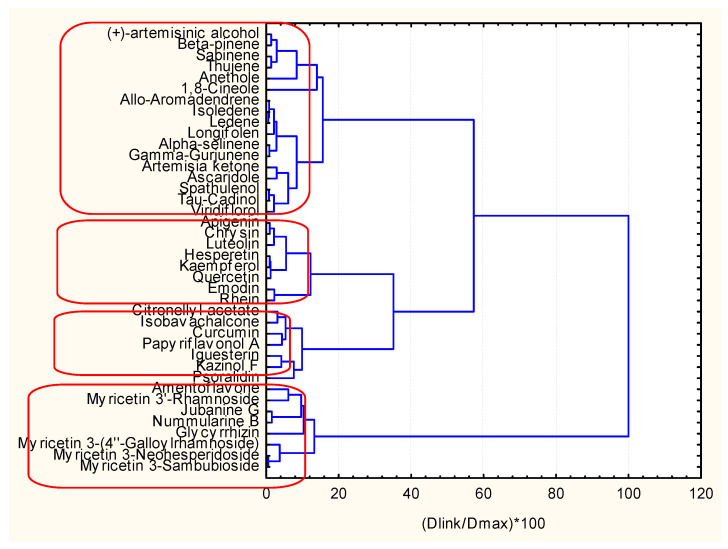
Hierarchical dendrogram for clustering of 40 objects.

**Figure 15:**
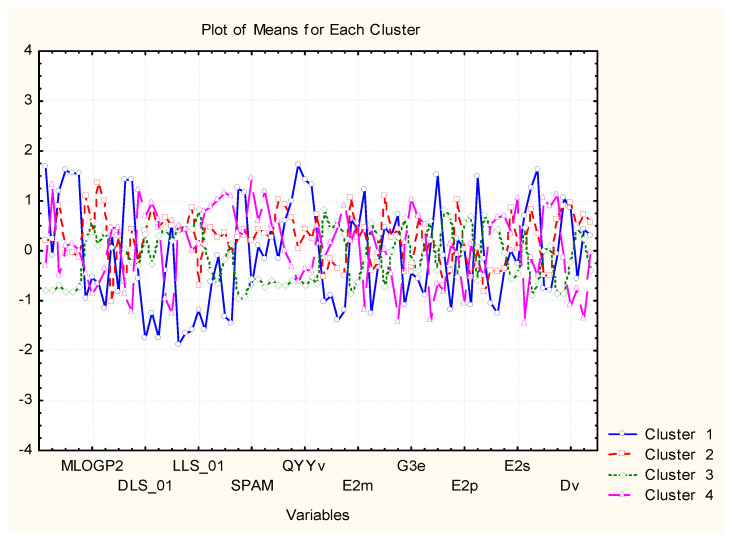
Plot of average (standardized) values of each variable for each identified cluster of objects.

The clustering by hierarchical mode and K-means (Figure 14 and Figure 15) gave the same results (the only difference is the numbering of the clusters if in the dendrogram one starts numbering from down to the top as C1, C2, C3, C4, the K-means clustering is C1, C2, C4, C3 with the same members.

Cluster 1 (different from the other three similarity patterns) is characterized most typically by the highest values for MW, RBN, TPSA, HBA, HBD, QXXv, QYYv, L2p, P2s, and lowest values for DLS_01, DLS_02, DLS_03, DLS_06, DLS_07, DLS_conc, LLS_01, LLS_02, CMC_50, CMC_80.

C2 is marked mainly by the highest values of ALOGP2 and LOGP99 but, in general, is in the intermediate position. The same holds true for C3, and the most specific feature for it is the lowest levels of MW, Mp, RBN, TPSA, HBA, HBD, MEcc, SPH, PJI3, L/Bw, DISPm, DISPv.

C4 is, to some extent, opposite in relation to descriptor values to C1. For instance, it is characterized by the highest levels of LLS_02, CMC_50, CMC_80, QED, QEDu, OXXv, OYYv.

Therefore, it is possible to obtain initial information about the clustering of objects, descriptors, and the mutual relationships between them. This preliminary step in the classification helps in future better interpretation of the classes of objects and variables formed.

### 2.9. Comparisons of Both Classification Results

Although the classification by the traditional classification methods offers 12 classes and four clusters analyses, there is a good coincidence between both approaches. In the hierarchical cluster, C1 includes classes 1, 3, 6, 11, in C2–2, 9, in C3–4, 7, 10, 12, and C4–7, 8. The finer partitioning in Table 3 is based on the mean docking score, but, in general, the partitioning is the same as the descriptors used in cluster analysis.

The comparison with the descriptor ranks (loadings) grouped in latent factors PC1 and PC2 shows that the grouping with respect to the rank is related to the partitioning of the descriptors into three clusters by cluster analysis: in PC1, there are members of the clusters 1 and 3 (cluster analysis), and in PC2, members of cluster 2 (except for the members of C1 MAXDN and MAXDP).

This relationship of both classification approaches allows using the patterns (class) formation results to solve other problems like docking operations. This classification technique could be used to identify another type of inhibitors. These classification techniques could accelerate the process of extensive screening making a preselection of suitable candidates.

### 2.10. Principle Component Analysis Self-Organizing Map Results

**Figure 16:**
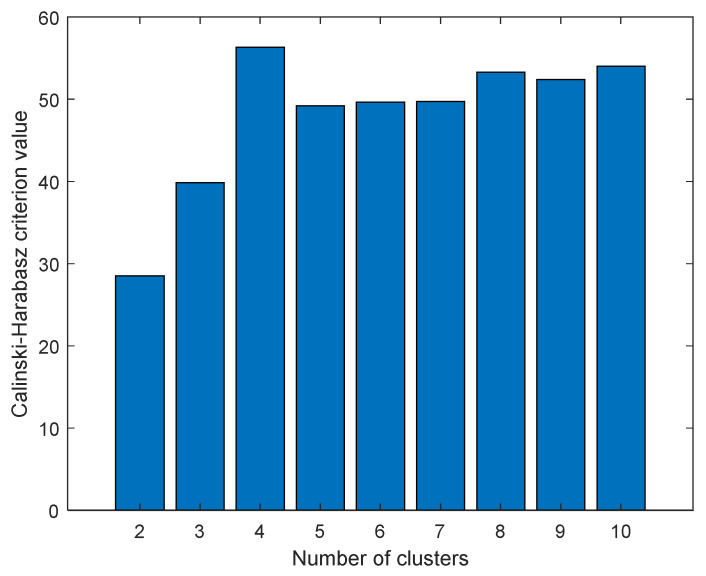
Calinski–Harabasz criterion values at different number of clusters (K).

The data were first scaled to centered on origin and have unit variance for all variables to investigate any possible similarity between compounds and find distant patterns. Next, PCA was applied to reduce the dimensionality of data, and K-means clustering method was then applied on the abstracted sample space. The number of latent factors in PCA can control the amount of variation which PCA can model. We used the first three components of PCA which explain almost 65 percentage of variance in data. The best number of clusters for K-means was based on Calinski–Harabasz (CH) criteria, which is the ratio of the sum of between-clusters and inter-cluster scatter for all clusters. Higher values for this criterion mean the clusters are well separated and dense. Hence, the higher the score, the better the performance. The CH criterion values were checked at different clusters (K) and the best value was K = 4 (Figure 16). Each compound in the score plot was depicted as a circle labeled with corresponding docking score in three-dimensional space. Figure 16 shows the PCA score plot for first three components. Different colors also specify the four clusters found by K-means clustering. To inspect the possible relation between compounds’ pattern and docking scores, each sample on the score plot is also labeled with a corresponding docking score value.

**Figure 17:**
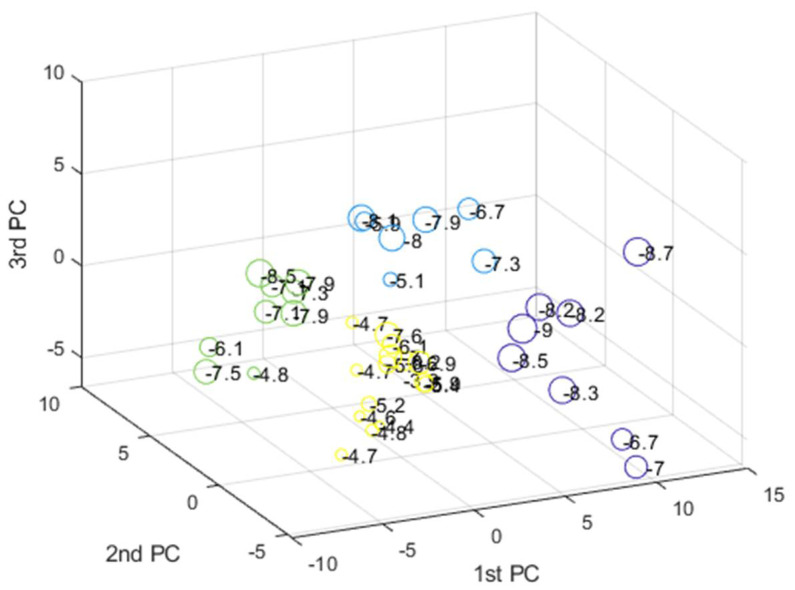
The PCA 3D score plot labeled with corresponding docking score values. Member of each cluster found by K-means clustering are depicted with different colors.

According to the clustering result and the pattern of compounds in the 3D score plot (Figure 17), members of each cluster have nearly similar docking score values, and the groups are well separated. Furthermore, most of the compounds with different docking score (high/low) are discriminated along the first PC direction.

**Figure 18:**
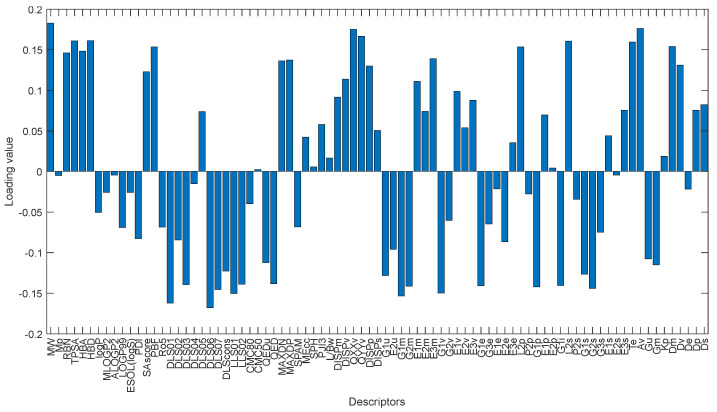
Bar plot of the loading values of 1st PC.

The first loading values defined by PCA can show the contribution of each variable to determine the first latent feature. As stated before, compounds with high and low docking score are well discriminated along first PC direction. Considering only the first loading, we can find the molecular descriptors that play an essential role in binding score of the compounds. Figure 18 shows the loading values for first PC. The molecular weight (MW) descriptor, quadrupole x-component value (QXXv), A total size index (Av) and modified drug-like score from Rishton (DLS_06) and quadrupole y-component value (QYYv) are the top five descriptors that contribute to define the first PC and discriminate cluster 1 (dark blue) from the others. These descriptors have a reasonable correlation with the reported docking score values. The MW, QXXv, QYYv and Av mean values for the cluster 1 are more significant than other groups and are positively correlated with the docking score. The mean value of the DLS_06 descriptors is negatively correlated with the mean docking score values of the clusters. 

### 2.11. Self-Organizing Map Results

**Figure 19:**
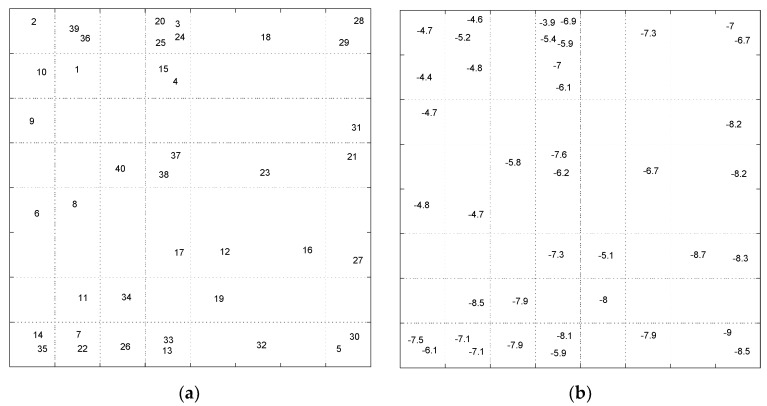
The Top-Map of SOM. (**a**) winner neurons for samples are labeled with compound number; (**b**) winner neurons are labeled with the docking score values.

Exploring the data topology in mapped feature space was also performed using Self-Organizing Map as a nonlinear dimensionality reduction method. The resulted maps from SOM can investigate the similarity between the compounds in feature space and any possible relation between the patterns and molecular descriptors. In this way, the scaled data matrix (40 × 83) was introduced to an 8 × 8 SOM row-wise. At the end of the training phase, molecules are mapped on the neurons such that similar molecules map on adjacent or the same neurons. The map was labeled using additional information such as row number or the docking. The resulted Top-Map of the trained 8 × 8 SOM labeled with sample number and binding score values are shown in Figure 19.

**Figure 20:**
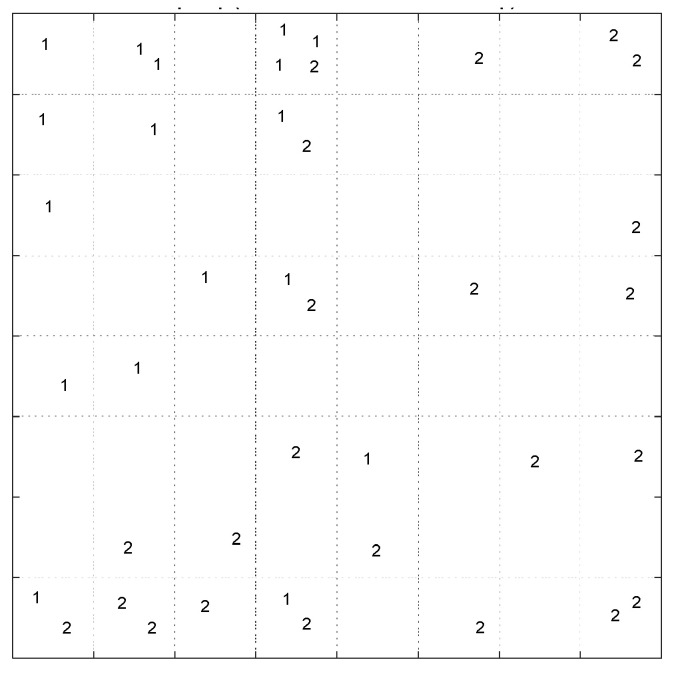
The Top-Map of SOM labeled with low (1) high (2) binding score.

The pattern of samples on the Top-Map (Figure 19a) shows similarities between compounds. Based on the molecular descriptors used to represent the compounds, highly similar molecules are mapped on the same or adjacent neurons. In addition, by considering the docking score of the molecules to label the sample (Figure 19b), we can conclude that most of the compounds mapped on the same or adjacent neurons have almost similar binding affinity. More precisely, the compounds mapped on the top-left side have less binding affinity than other map parts. The SOM algorithm can distinguish the molecules with different binding affinities. Considering the range of binding scores for all of the molecules, we divided the molecules into two groups: low binding score (>−6.45) specified with label ‘1’, and high binding score (≤−6.45) which specified by label ‘2’ on the following Top-Map (Figure 20).

**Figure 21:**
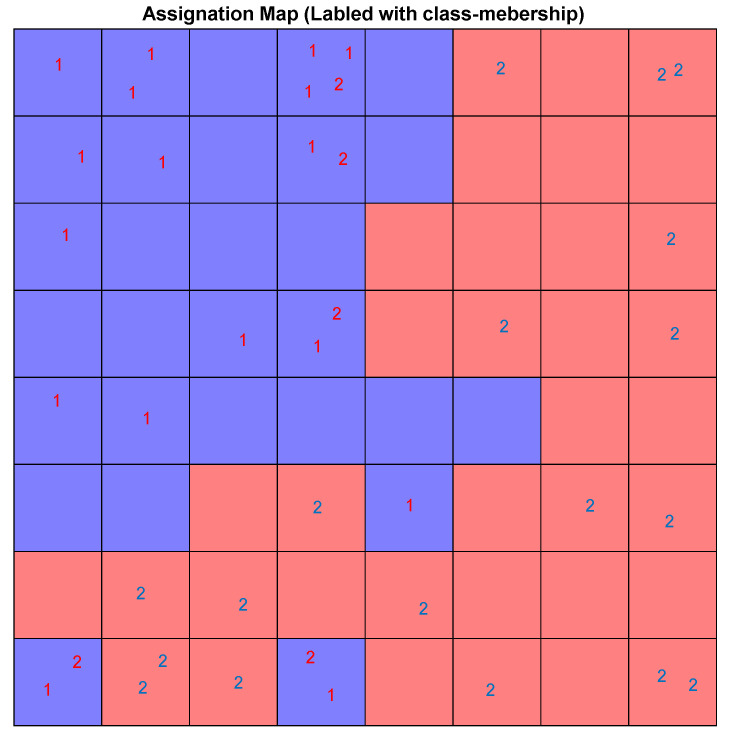
The assignation map of CPANN labeled with low (1) high (2) binding score. The neurons are colored in red or blue based on output layer of the network. The red area is assigned to high docking score compounds and the blue regions.

Considering the low/high docking score as a class membership label makes it possible to use the supervised version of SOM, the counter-propagation artificial neural network (CPANN). CPANN can produce a Top-Map similar to a classical SOM and assign a class to each neuron that can be used to predict labels for unknown samples classification purposes. The size of the map was decided based on a Genetic Algorithm-based optimization method included in the Kohonen and Counter Propagation Toolbox for MATLAB. The best architecture and setting for the network is an 8 × 8 grid and 250 training epochs. Figure 21 shows the assignation map obtained using an 8 × 8 CPANN trained with the data and class information.

**Figure 22:**
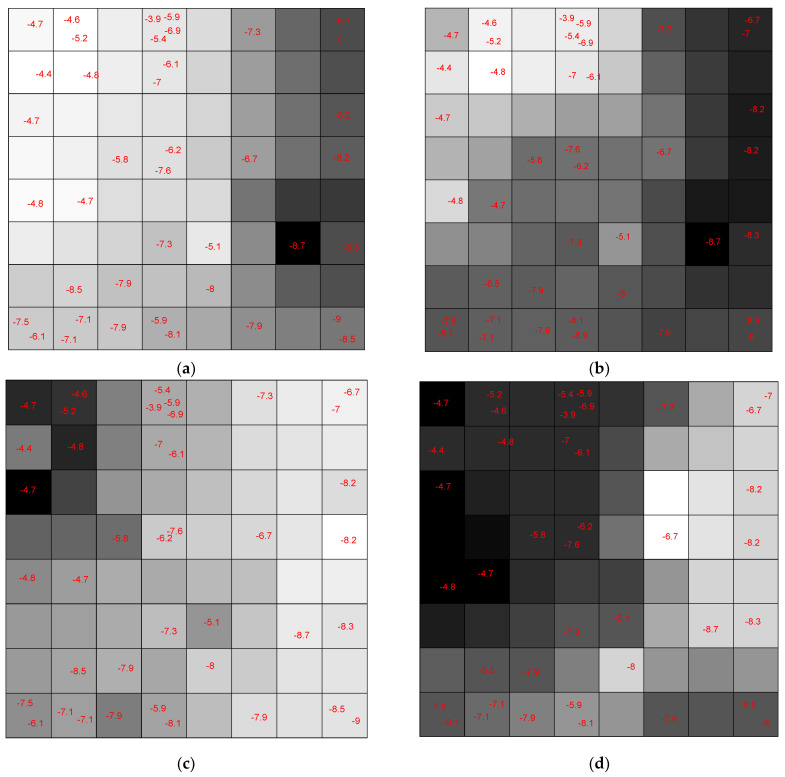
The Weight Map of (**a**) molecular weight (MW) descriptor; (**b**) maximal electrotopological positive variation (MAXDP) descriptor; (**c**) 1st component symmetry directional WHIM index weighted by Sanderson electronegativity (G1e) descriptor; (**d**) modified lead-like score from Congreve (LLS01) descriptor.

Each neuron has an input weight vector that was updated during the training phase of the CPANN. Considering only the *i*th weight vector elements of all neurons, *i*th weight map can be obtained by shading the neurons. Comparing these weight maps to the pattern of the assignation map, we investigated the correlation of each variable with the assignation map. We found the most influential variables in the mapping process. The weight maps of several descriptors are shown in Figure 22. The darker shade means a higher value for the corresponding weight element and vice versa.

**Figure 23:**
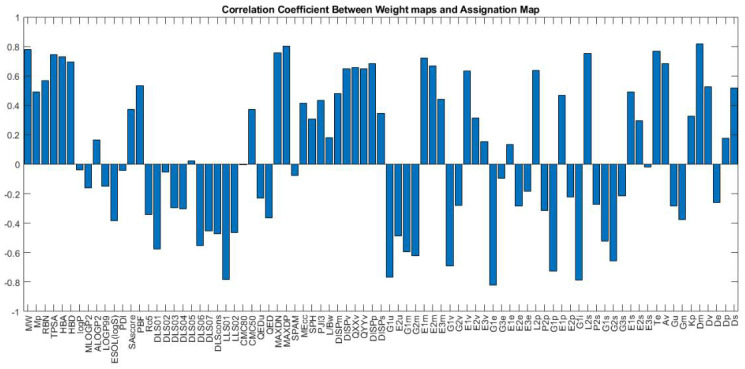
The correlation coefficient between each weight-map (corresponding to each descriptor) with the assignation map.

Figure 23 shows the weight maps of some important variables. It is apparent that the pattern of the weight map for the MW descriptor is positively correlated with the pattern of classes in the assignation map, and we can conclude that the molecular weight is an important descriptor related to the docking score and the molecules with higher molecular weight have a higher binding score. The maximal electro-topological positive variation (MAXDP) descriptor is also another variable that is highly correlated with the pattern of the docking score in the assignation map. In order to have better insight into the importance of variables, the Pearson correlation coefficient between the weight map of each variable and the assignation map is calculated and summarized in Figure 23.

As shown in the figure, MW, TPSA, HBA, HBD, MAXDN, MAXDP, and DM are the most important descriptors, with a positive correlation with binding scores. Among the descriptors with the negative correlation, the most correlated ones are G1e, LLS01, G1u, G1i, and G1p. The weight maps of G1e and LLS01 are also shown.

## 3. Material and Methods

Identification of features of natural compounds required for higher druggability, using ML techniques.

### 3.1. Principle Component Analysis (PCA)

PCA is one of the most common multivariate data analysis techniques used for compression and dimensionality reduction. [25]. Each molecule can be represented as a vector in Principle Components (PC) space, and this plot can be used to investigate similarities and patterns in sample space [26]. In addition, by plotting the columns of loading matrix P, the loading plot can be obtained. Each variable can be represented as a vector in the reduced space defined by PCs. The loading plot can be used to investigate the contribution and importance of the variables to specify PC directions and find possible patterns and similarities between variables.

### 3.2. K-Means Clustering

Clustering algorithms play an important role in data mining tasks such as partitioning data samples into subsets or categories. One of the most common clustering methods is the K-means clustering [27]. K-means as a non-hierarchical method divide the samples into clusters based on a similarity measure like Euclidean distance and the descriptive variables used to represent samples. The number of clusters (K) should be decided before starting algorithm. In this work, it was determined using Calinski–Harabasz criterion [24], which considers the ratio of the sum of between-clusters scatter and of within-cluster scatter for all clusters. The higher ratio can be concluded as a proper number of clusters and centroid positions.

### 3.3. Self-Organizing Map (SOM)

The Self-Organizing Map (SOM) algorithm [28] is a neural network based on competitive learning which can be used for data visualization, and nonlinear dimensionality reduction in an unsupervised manner [29]. It can ease in visualizing the structure of the data in high-dimensional feature space while preserving the data topology mapped onto a two-dimensional grid of neurons which make the SOM suitable for finding clusters and complex patterns [30]. The classical SOM consists of a 2-D grid of neurons organized on a regular low-dimensional grid. Each neuron is associated with a weight vector that has the same dimension as the input vectors. The training algorithm of SOM manages the mapping process to place similar input vectors on the same or adjacent grid position as well as the dissimilar ones on distant grid positions. Training phase of the SOM consists of two main steps: (i) The competitions step in which the winner neuron was decided based on the similarity of the input vector and weight vector of all neurons on the grid. The Euclidean distance is the most common similarity measure used to find the winner neuron; (ii) The cooperation step in which the weight vectors of the winner neuron and its neighboring neurons are adopted by the following equation:

$$w_{i}\left(t+1\right)=w_{i}\left(t\right)+LR\left(t\right)\times h\left(t\right)\times \left(x_{j}-w_{i}\left(t\right)\right)$$

where $w_{i}$ is the weight vector of *i*th neuron, *t* is the current time, *LR* is the learning rate and *h* is the neighborhood function which could be a radial function like Gaussian. The width of the neighborhood function and the learning rate are reduced during the weight adaptation procedure. In this way, the weight vector of the winner and its neighbor neurons become more similar to the presented input vector. In this way, similar input vectors will be placed on same or adjacent zones of the grid. After the completion of the training, the Top-Map can be obtained by labeling the winner neuron of each input vector with a proper information. Top-Map can help to visualize and distinguish any similarity between input vectors and finding patterns in the high dimensional feature space in an unsupervised scheme. In order to incorporate label information in training of SOM, Counter Propagation artificial neural networks (CPANN) as a supervised variant of SOM were proposed. CPANN consists of two layers of neurons to handle both input and corresponding class vector in a training algorithm similar to SOM. After training, it can produce a top-map similar to a classical SOM. The input and output weight vectors of the CPANN neurons have the same dimensions as input and label vector respectively. Considering the weight vectors of the neurons in the output layer of a trained network, a class membership can be assigned to each neuron position to form an Assignation-Map. This map can help to reveal complex class structures as well as similarity between input vectors in a supervised manner.

By considering corresponding weight vector elements of all neurons (Weight Map) and comparing them to the pattern of the assignation map, we can investigate the correlation of each variable with the assignation map and can find the most effective variables in mapping [31,32].

## 4. Conclusions

A combination of molecular docking, MD simulations and ML techniques have been applied to identify natural compounds and their features that could substantially bind to the spike protein of SARS-CoV-2 and disrupt its interaction with ACE2 receptor. Binding affinity and dynamic behavior of 40 phytochemicals were examined against the X-ray crystal structure of the SARS-CoV-2 S1-RBD (PDB ID: 6M0J). We identified four potential binding sites in the protein structure. Molecular docking of the ligands to these sites show high binding affinity for amentoflavone, glycyrrhizin, chrysin, myricetin_3’-Rhamnoside and myricetin_3-(4″-Galloylrhamnoside). Molecular dynamic simulation of selected protein–ligand complexes shows a long retention time of Amentoflavone in pocket 1 of the spike protein, and the interaction is mainly driven by H bond interactions. Ligand Glyrimizine mainly binds at pocket 3 and MYG binds at pocket 1A. Based on binding affinity results and retention time, we conclude that Amentoflavone and Glyrimizine can potentially disrupt the interaction between ACE2 receptors and the spike protein of SARS-CoV-2.

The features of best binding drugs were further identified based on classification schemes. Based on Self-Organizing Map analysis, the primary descriptors are MW, TPSA, HBA, HBD, MAXDN, MAXDP, and DM as essential descriptors for better binding with spike protein. The machine-learning model, docking and molecular dynamics studies can predict the new inhibitors based on a set of obtained molecular descriptors. The current workflow can be used for the identifying descriptors, followed by identification of features that are important for drug development against the spike protein of SARS-CoV-2.

## Figures and Tables

**Table 1:** Predicted binding sites in the receptor-binding domain.

Pocket	Area (Å^2^)	Volume (Å^3^)	Residues in Pocket
Pocket 1	73.389	60.837	PHE_338, GLY_339, PHE_342, ASN_343, ALA_344, THR_345, VAL_367, SER_371, SER_373, PHE_374, TRP_436, ASN_437, LEU_441, ARG_509
Pocket 1A	--	--	THR_333, ASN-334, LEU_335, CYS_336, CYS_361, VAL_362, ALA_363, ASP_364, TYR_365, SER_366, ASN_388, ASP_389, CYS_391, THR_523, VAL_524, CYS_529, GLY_526
Pocket 2	50.911	28.850	ARG_454, PHE_456, ARG_457, LYS_458, ASP_467, SER_469, GLU_471, TYR_473PRO_491
Pocket 3	40.366	15.858	GLU_340, VAL_341, ALA_344, ARG_346, PHE_347, ALA_348, SER_349, TYR_351, ALA_352, ASN_354, TYR_449, ASN_450, TYR_451, LEU_452, ARG_466, ASP_467, ILE_468, SER_469, THR_470, GLU_471, ILE_472

**Table 2:** Drug likeness properties of phytocompounds.

Name	Molecular Weight	Rotatable Bonds	TPSA	HBA	HBD	logP	MLOGP2	LOGP99	logS
Amentoflavone	538.48	3	174	10	6	5	0.49	18.98	−5.50
Glycyrrhizin	823.04	7	267	8	16	2.8	0.45	5.83	−5.52
Chrysin	254.25	1	67	4	2	3.5	6.51	6.76	−5.52
Myricetin_3′-Rhamnoside	464.41	3	207	12	8	0.5	3.99	0.04	−2.32
Myricetin_3-(4″-Galloylrhamnoside)	616.52	6	280	16	10	1.7	8.04	1.62	−3.17

Labels: LogP—lipophilicity, HBA—number of H bond acceptors, HBD—number of H bond donors, TPSA—total polar surface area in A2, lopP—partition coefficient octanol/water, MLOGP2—squared Moriguchi octanol–water partition coeff. (logP2), LOGP99—Wildmann–Crippen octanol–water partition coeff. (logP), logS—estimated solubility (logS) for aqueous solubility using LOGPcons.

**Table 3:** Molecular docking scores and related properties.

Drug Molecule	Affinity (kcal/mol)	Receptor’s Rgyr (nm)	Receptor’s SASA (nm^2^)	Ligand’s SASA (nm^2^)	System’s SASA (nm^2^)	Contact Area (nm^2^)	Surrounding Residues	Hydrogen-Acceptor and Hydrogen-Donor Distances
Amentoflavone	−9	1.80	103.83	7.66	104.80	3.34	LEU 335	1
							LEU 368	1
							CYS 336	1
Glycyrrhizin	−8.7	1.80	103.83	10.20	102.57	5.73	ASP 364	2
							TRP 436	2
							ARG509	2
							LEU 335	1
							PHE 342	1
							VAL 362	1
							ALA363	1
Chrysin	−8.5	1.80	103.86	4.54	103.08	2.66	PHE 338	1
							GLY 339	1
Myricetin_3′-Rhamnoside	−8.5	1.80	104.5506	6.8117	103.03	4.16	ASN 343	1
							VAL 367	1
Myricetin_3-(4″-Galloylrhamnoside)	−8.3	1.80	103.72	8.303	103.41	4.30	PHE 338	1
							ALA 344	1
							SER 371	1
							LEU 441	1
							ARG 509	1

## Data Availability

The Protein–ligand analyzer tool is freely available at https://www.samson-connect.net/element/98bd1552-4642-9e86-6a78-83c9e96a63ee.html (accessed on 15 April 2021) The in-home made code for PCA plotting is freely available at GitHub: https://github.com/mici345/PCA-MATLAB-R2019-Statistics-and-Machine-Learning-Toolbox- (accessed on 15 April 2021) with the data matrix representing the information of 40 compounds using 83 descriptors, and it is prepared in a readable format for MATLAB. Other data is contained within the article and Supplementary Materials.

## Supplementary Materials

The following are available online at www.mdpi.com/article/10.3390/ph14121328/s1, Table S1: Dataset of 40 natural compounds with docking scores and properties; Table S2: Complete dataset with a full list with descriptors; Figure S1: Molecular representations for the used ligands.
