# Supplementary material for: Inhibition Ability of Natural Compounds on Receptor-Binding Domain of SARS-CoV2: An In Silico Approach
Source: Pharmaceuticals (Basel). 2021 Dec 18;14(12):1328. doi: 10.3390/ph14121328 (PMC8704597; doi:10.3390/ph14121328)
Supplement: Supplementary file 1 [file pharmaceuticals-14-01328-s001.zip › pharmaceuticals-1496911/Figure S1.pdf]

Supplementary Information

Inhibition ability of natural compounds on receptor-binding domain of SARS-Cov2: An in silico approach

Figure S1: Molecular representations for the used ligands.

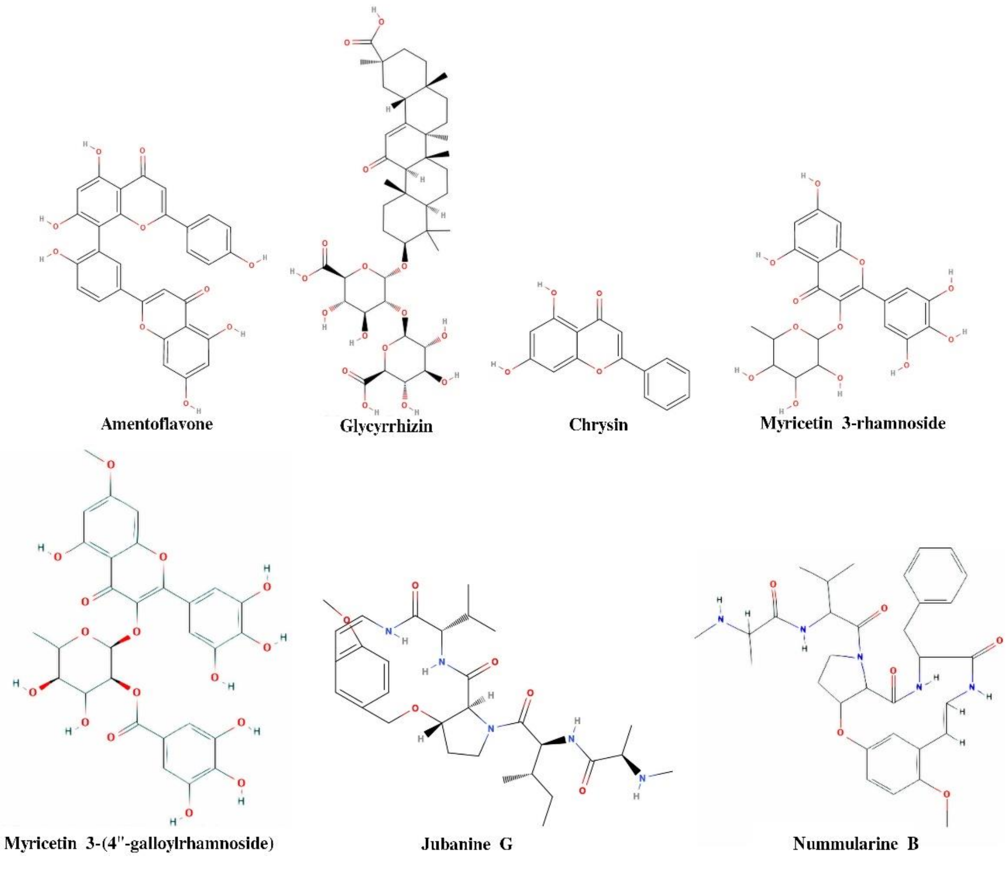

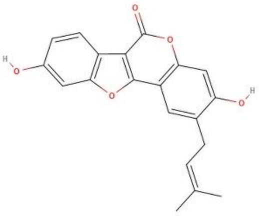

Psoralidin

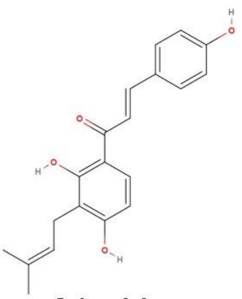

Isobavachalcone

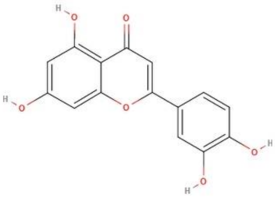

Luteolin

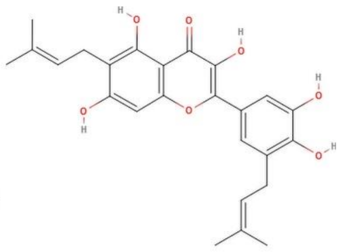

Papyriflavonol a

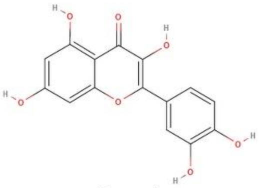

Quercetin

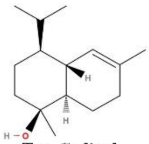

Tau-Cadinol

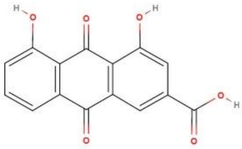

Rhein

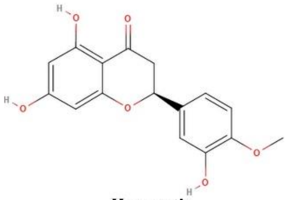

Hesperetin

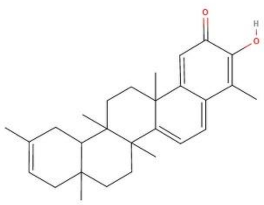

Iguesterin

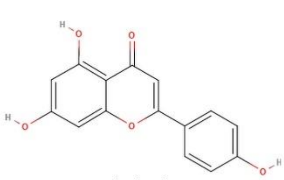

Apigenin

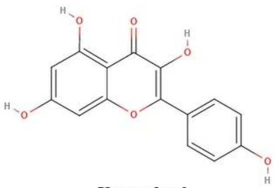

Kaempferol

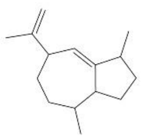

Gamma-Gurjunene

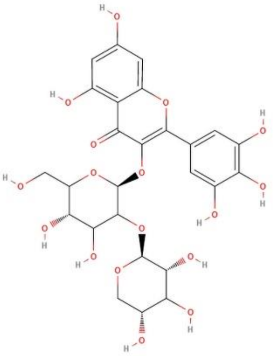

Myricetin 3-sambubioside

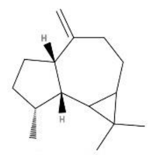

Allo-Aromadendrene

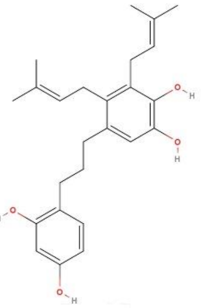

Kazinol f

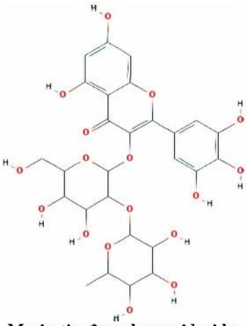

Myricetin 3-neohesperidoside

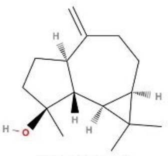

Spathulenol

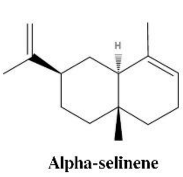

Alpha-selinene

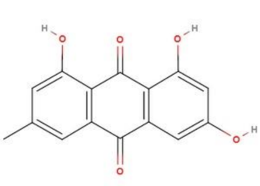

Emodin

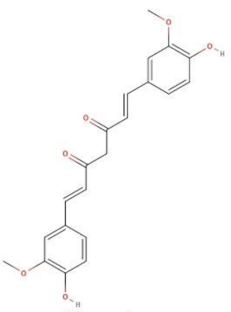

Curcumin

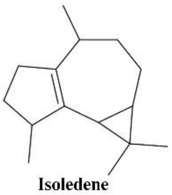

Isoledene

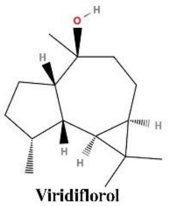

Viridiflorol

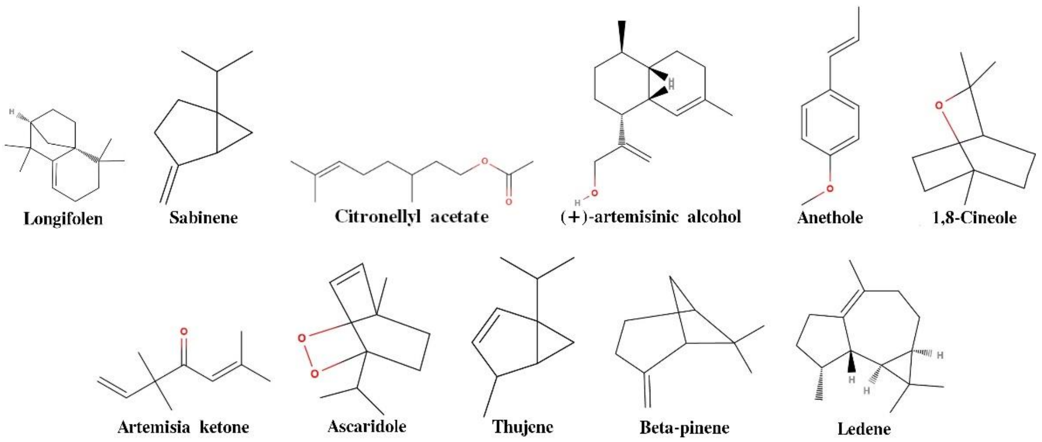
Longifolen
Sabinene
Citronellyl acetate
(+)-artemisinic alcohol
Anethole
1,8-Cineole
Artemisia ketone
Ascaridole
Thujene
Beta-pinene
Ledene
